# An international breeding project using a wild potato relative *Solanum commersonii* resulted in two new frost-tolerant native potato cultivars for the Andes and the Altiplano

**DOI:** 10.3389/fpls.2024.1358565

**Published:** 2024-03-05

**Authors:** Jesus H. Arcos-Pineda, Alfonso H. del Rio, John B. Bamberg, Sandra E. Vega-Semorile, Jiwan P. Palta, Alberto Salas, Rene Gomez, William Roca, David Ellis

**Affiliations:** ^1^ Instituto Nacional de Innovacion Agraria (INIA), Estacion Experimental Agricola (EEA) Illpa-CE Salcedo, Puno, Peru; ^2^ U.S. Department of Agriculture (USDA)/Agricultural Research Service, Potato Genebank, Sturgeon Bay, WI, United States; ^3^ Department of Plant and Agroecosystem Sciences, University of Wisconsin—Madison, Madison, WI, United States; ^4^ International Potato Center (CIP), Lima, Peru

**Keywords:** andean potato cultivars, climate change, benefit sharing, frost tolerance, genetic diversity, potato wild relatives, *Solanum commersonii*, U.S. Potato Genebank

## Abstract

This breeding project, initiated at the United States Potato Genebank (USPG) in collaboration with Peruvian partners Instituto Nacional de Innovacion Agraria (INIA), International Potato Center, Peru (CIP), and local farmers, sought to enhance cold hardiness and frost tolerance in native potato cultivars in Peru. The Andes and Altiplano are often affected by frost, which causes significant reduction in yield; creating varieties with superior resilience is a critical undertaking. The goal was to transfer outstanding non-acclimated cold tolerance and acclimation capacity found in wild potato species *Solanum commersonii (cmm)*. Breeding families segregating for cold hardiness were created using (a) a somatic hybrid *cmm* + haploid *Solanum tuberosum* (*tbr*) (cv. Superior, US variety from Wisconsin) as male and (b) seven cultivars native to Peru of the species *S. tuberosum* sbsp. *andigenum* (*adg*) as females. All plant materials were part of the USPG germplasm collection. Sexual seeds of each family were sent to Peru for evaluations under the natural conditions of the Andean highlands and Altiplano. The plants were assessed for their response to frost, and genotypes showing exceptional tolerance were selected. Plants were also evaluated for good tuber traits and yield. Initial planting involving ~2,500 seedlings in five locations resulted in selecting 58 genotypes with exceptional frost tolerance, good recovery capacity after frost, and good tuber traits. Over the years, evaluations continued and were expanded to replicated field trials in the harsher conditions of the Altiplano (Puno). All trials confirmed consistency of frost tolerance over time and location, tuber quality, and yield. After 8 years, two advanced clones were considered for cultivar release because of their exceptional frost tolerance and superior field productivity that outyielded many of the established cultivars in the region. In November 2018, a new native cultivar named *Wiñay*, a Quechua word meaning “to grow” was released in Peru. In 2022, a second cultivar followed with the name *Llapanchispaq* (meaning “for all of us”). This project evidenced that a multinational and all-encompassing approach to deploy valuable genetic diversity can work and deliver effective results. This is even more significant when outcomes can promote food security and sustainability in very vulnerable regions of the world.

## Introduction

In the Andes and the Altiplano of Peru and Bolivia, frost is responsible for serious damage to agriculture affecting many smallholders in a situation of high vulnerability, i.e., subsistence potato farmers in conditions of poverty or extreme poverty (Condori et al., 2014). Approximately 74% of the agricultural communities there are exposed to frost (Hijmans, 1999). Though the damages can be irreversible, potato farmers try to counteract these impacts through the ancient tradition of planting mixtures of many native cultivars expecting that some can survive and produce. For example, cultivars of cultivated potato species *Solanum juzepczukii* and *Solanum curtilobum* have excellent cold hardiness and are part of the farmers’ cultivar pool. However, their tubers are bitter due to high glycoalkaloid content and cannot be eaten fresh unless the ancient freeze-drying processes of *chuño* and *tunta* are used to remove alkaloids (Brush et al., 1981). It is estimated that approximately 25% of all potatoes in the Altiplano are bitter; hence, adding frost-tolerant non-bitter cultivars could be a very significant contribution. In that way, farmers can consume the tubers and/or place them in regional markets immediately with no need for additional processing to eliminate bitterness.

Screening and identifying crop germplasm expressing genetic traits with enhanced resilience to abiotic stresses is especially relevant today. The impact of climate change in agriculture is serious and responsible for food shortages and economic distress (Morton, 2007). The possibility of incorporating valuable genetic diversity to create new varieties with enhanced adaptation to new climates must be considered a strategy to prioritize. Potato germplasm, which includes the potato crop and its wild relatives, is an outstanding source of genetic resistance to different pests and diseases and to extreme tolerance to abiotic stresses (Li and Palta, 1978; Palta and Li 1979; Hanneman, 1989; Tiwari et al., 2022). In particular, the wild relative species *Solanum commersonii (cmm)*, endemic to Uruguay, Argentina, and Brazil, possesses outstanding freezing tolerance and ability to cold acclimate—two important genetic traits that, if transferred to cultivated potatoes, can make a large impact in resilience to low temperatures and frost (Li and Palta, 1978; Palta, 1991; Stone et al., 1993; Vega and Bamberg, 1995; Aversano et al., 2015; Cho et al., 2016). Every year, the potato experiences major yield losses worldwide due to frost (Li and Palta, 1978; Palta and Li 1979). The good news is that major increases in frost tolerance are not needed to have significant impacts. Hijmans et al. (2003a, b) estimated that if cold tolerance only increases by 1°C or 2°C, potato yields can be improved by 26% and 40%, respectively.

Because of its outstanding cold hardiness, germplasm enhancement and breeding programs investigated the possibility of using *cmm* to enhance freezing tolerance. However, it was found that some wild potato species, including *cmm*, are not possible for use in crossbreeding because of their reproductive incompatibilities. Johnston et al. (1980) reported that inter-specific crosses between *cmm* and cultivated potatoes failed because of post-zygotic barriers. This resulted in investigating other options to overcome cross incompatibility. Hence, it was discovered that techniques of somatic fusion of leaf protoplasts were effective to overcome incompatibility and combine the genes of otherwise incompatible species. The fact that potatoes can be clonally propagated was important because all useful gene combinations established in the somatic hybrids were maintainable. Mattheij and Puite (1992) and Millam et al. (1997) showed that methods of protoplast fusion and somatic hybridization were effective in generating viable somatic hybrids between *cmm* and other incompatible species. Moreover, Cardi et al. (1993) generated *cmm + tbr* hybrids with improved levels of frost hardiness and acclimation capacity proving that introgression of *cmm* was effectively attained and expressed. Later, field evaluations of selfed and backcrossed progenies derived from somatic hybrids *cmm* + *tbr* were conducted in the US, which resulted in finding lines with excellent tuber quality and production (Millam et al., 1995; Chen et al., 1999a, b, c). This unlocked the idea of testing them in Peru with the expectation that their remarkable cold hardiness and productivity could be replicated in the Peruvian highlands. However, they gave poor yields as they were not adapted to the shorter daylength of the Andes (because of the *tbr* background). That setback led to a new plan of developing breeding lines for cold hardiness in Peru, but this time utilizing the well-adapted native cultivars of *adg* as the parental lines.

Here, we present the results of an international breeding project initiated at the USPG that examined the prospects of using the potato wild relative *cmm* to transfer enhanced cold hardiness, cold acclimation capacity, and frost tolerance into potato cultivars of *S. tuberosum* subsp. *andigenum*, the potato cultivated in the Andean and the Altiplano regions. From a broader perspective, this project was a model of international cooperation to promote participatory work and share benefits in the utilization of genetic resources.

## Materials and methods

### Development of frost-tolerant breeding families

The parental materials used to generate the breeding families were (1) a somatic hybrid between *S. commersonii* + a haploid of *S. tuberosum* cv. Superior (USW-13122) developed in the Wisconsin breeding program (Chen et al., 1999a; Kim-Lee et al., 2005) and (2) seven native cultivars of the species *S. tuberosum* ssp. *andigenum* (*adg*) as female parents (**Table 1**). The somatic hybrid *cmm + tbr* is a genetic stock maintained in the USPG collection as accession number GS393. The accessions used as female *adg* parents were not the original cultivar clones, but the sexual progeny (seed lots) derived from them and maintained at the gene bank as botanical seeds. All plant materials were part of the USPG germplasm collection in Sturgeon Bay, Wisconsin, USA.

**Table 1:** List of germplasm used as parents to generate frost-tolerant families at the USPG.

Family Code	Female parent	Local name of the native cultivar (if reported in GRIN database)
	USPG PI numbers*	
**H1**	281078	
**H2**	281065	
**H3**	292086	*Ccompis*
**H4**	281070	
**H5**	308886 x 308885	*Color uncuna x Chaquillo*
**H6**	246555	*Suytta*
**H7**	281063	

Some of the *adg* accessions have a local cultivar name in Peru listed in the database and all originated in Cusco, Peru. The same male parent (somatic hybrid *cmm* + *tbr*; PI number GS393) was used to generate each family.

*Additional information is available through the Germplasm Resources Information Network (GRIN) https://npgsweb.ars-grin.gov/gringlobal/search.

The F1 progenies generated were then subsequently bulk intermated yielding F2 populations. To estimate potential cold hardiness, assorted genotypes from the different families were assessed for relative freezing tolerance (RFT) in non-acclimated and acclimated stages. RFT was estimated in the laboratory by measurements of ion leakage of excised terminal leaflets subjected to ice nucleation and simulated freeze–thaw stress (Li and Palta, 1978). RTF values in non-acclimated genotypes ranged from −2.4°C to 4.0°C, while in acclimated stage, they ranged from −3.0°C to −5°C. Seeds of these seven breeding families expected to segregate for freezing tolerance were sent to Peru in 2009 to be evaluated for their frost response under field conditions in the Andes and the Altiplano.

### Selection of genotypes expressing frost tolerance

#### Field experiments

The initial field trial in 2009–2010 included approximately 2,500 randomly selected seedlings, which were a proportional share of several genotypes from each of the seven families. Plants were initially grown in greenhouses at the International Potato Center, Peru (CIP) in Lima, Peru. When the plants were approximately 15 cm, they were transported by car to the different fields in the highlands.

The distances to the fields from the city of Lima were between 300 and 1,000 km. Fields were in farmers’ communities in the highlands, in the towns of *San Jose de Aymara*, *Huancavelica* (−12.243, −75.051; 3,906 m); *Aramachay*, *Junin* (−11.916; −71.413; 3,687 m); *CCorao*, *Cusco* (−13.478; −71.923; 3,596 m); and *Kayra*, *Cusco* (−13.541; −71.888; 3,426 m).

All fields were rainfed except for *Kayra*, which is an experimental field in the University San Antonio de Abad in Cusco. In addition, the fields were in areas where freezing temperatures and unexpected frosts are common during the growing season. As this project promoted participatory work, the activities of land preparation, planting, hilling, selection, and evaluations were done with the help of local farmers at each community.

### Assessment of plant damage to determine levels of frost tolerance

One week after the frost event, the fields affected were visited to assess the levels of damage in the plants and to estimate the survival rates. This 1-week waiting made more evident the effects of frost on the plants and facilitated the assessments and ratings. Symptoms of freezing damage in the plants ranged from minor ones, like darkening of the leaf margins, to more serious ones, like wilted and/or dead leaf and stem tissue. The magnitude of leaf and whole-plant injury was determined by visual estimations and reported as percentage of plant damage.

After 2 to 3 weeks, the affected fields were visited for a second time to assess the levels of plant recovery. At the end of the season, the genotypes rated with good levels of frost tolerance and ability to recover (and desirable tuber features) were selected for the next round of field trials. For field trials, the genotypes were coded with the acronym HSP, which stands for H=frost (as for the Spanish word *heladas*), S=row number (Spanish *surco*), and P=plant number in the row (Spanish *planta*). The number after H identified the family where the genotype originated (see **Table 1**).

### Seedling selection and clonal generations

Seedling selection aimed to identify genotypes with superior tolerance to frost. Because of the *adg* background, the breeding lines were anticipated to be adapted and respond well to the traditional practices in the Andes. Adaptation implied plants with short-day tuberization and ability to grow in soils with low fertility and low pH. After seedling selection, clonal generations were generated for the following season of 2010–2011 using the tubers harvested as seeds. This sought to confirm if selections were consistent in exceptional frost responses and the expression of desirable tuber traits. Trials of clonal generation selections involved larger number of plants in the field, so the results were more representative as sampling variation was included in the analyses. Hence, the experimental field trials consisted of tuber seeds in single rows with up to five plants per clonal line. The same methods to assess plant injury, indicated above, were used to determine levels of frost damage in the plants. Yield was measured as tuber number and weight. Farmers from the local rural communities nearby participated in the evaluation and selection process.

### Advanced selections and evaluations for cultivar release

Field trials for advanced selections were carried out under the harsher conditions found in experimental fields in Puno, in the Altiplano region, where crops are constantly negatively affected by environmental stresses. Gilles and Valdivia (2009) pointed out that because of frost and other abiotic stresses in the Altiplano, the potato never reaches its full yield potential. The experimental fields were in *Salcedo* (−15.868, −69.995, 3,838 m) and *Illpa* (−15.832, −70.019, 3,815 m). Both are part of the System of Agricultural Stations for Peru’s National Program for Agriculture (INIA).

The advanced selections were planted together with other common native cultivars of the region as comparisons. All were evaluated in randomized complete block designs with up to five replicates per location. Plots consisted of single rows of up to 10 plants spaced at ~0.60 m between plants. At harvest, tubers of each plot were counted and weighed to determine the averaged tuber yield. Throughout the different years of field trials, the popular cultivars of the Altiplano region used were *Imilla Blanca*, *Imilla Negra*, *Ccompis*, *Mariva*, *Pucamama*, *Lekecho*, and *Oke* (Cahuana-Quispe and Arcos-Pineda, 2002).

In the final step, the advanced clones identified as potential new cultivars were validated under the INIA guidelines for cultivar release stated under Peru’s National Policy number 047-2000-INIA (El Peruano, 2000). The guidelines indicate the procedures needed to test the potential cultivars for parameters a new plant variety must meet, namely, distinguishability, homogeneity, stability, validation, and homologation,. The guidelines can be found in https://www.fao.org/faolex/results/details/en/c/LEX-FAOC020078.

The validation approach expected multilocation field trials of the potential cultivars, which must be grown with local cultivars as controls. These trials must be done in at least 10 different farmers’ communities in the region, and then, surveys must be conducted in a subset of the population at each community to confirm the support on accepting these clones as new cultivars for the region.

### Statistical analysis

The results of the field evaluations for frost and yield followed an analysis of variance using JMP Pro 15 statistical software to get additional insights on the levels of differential responses among genotypes. A Tukey–Kramer LSD test was used to determine the significance of the differences between mean values at a probability level *p* ≤ 0.05 (JMP® Pro, Version 15.0.0, 2019).

## Results

### Detection of genotypes expressing good levels of frost tolerance

**Figure 1:**
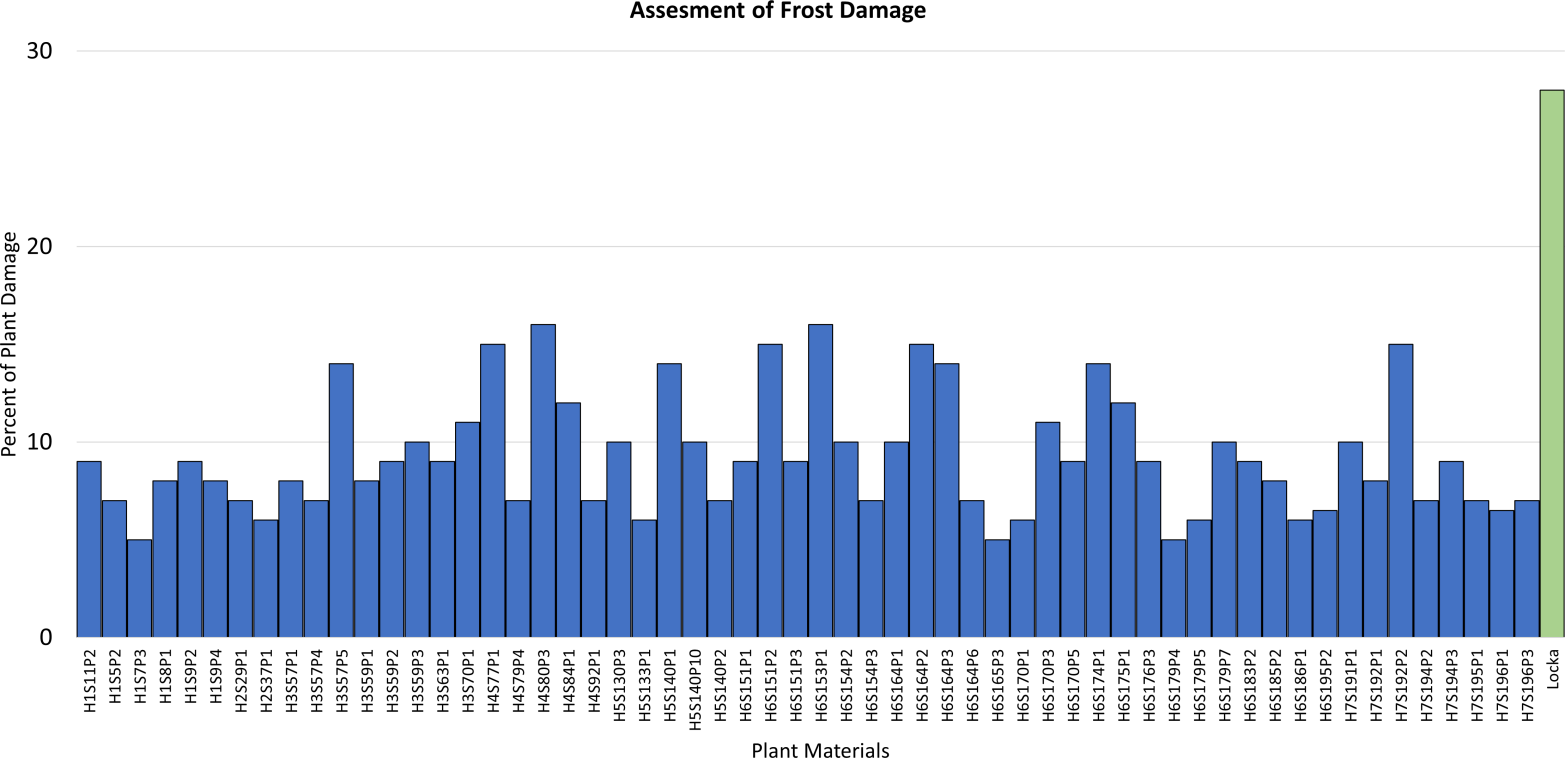
Seedling evaluations of frost damage in the fields showing the 58 genotypes of the cold breeding families identified with exceptional levels of frost tolerance (measured as percent of plant damage). The native cultivar *Locka* was included as comparison.

After a frost, fields were assessed to determine damage to seedlings. The extent of damage observed in the fields ranged broadly from the total loss of the plant to a minimum plant damage of ~5%. In general, frost episodes in that season were in the range of −2°C to −5°C. Only the genotypes with plant damage under 20% were considered for selection, and that produced a total of 58 genotypes. All of them showed plant damage levels under 20% and expressed recovery ability after a frost rated as either good or very good. Assessments for plant damage after a frost for those 68 selections are presented in **Figure 1**. To get an idea of the levels of cold hardiness, these genotypes were compared to a native potato cultivar of the region named *Locka.* This is a cultivar of the species *S. jusepczukii* characterized by bitter tubers but cold hardy and adapted to the Altiplano (Cahuana-Quispe and Arcos-Pineda, 2002). Though evaluations at harvest were limited to only one plant, the 58 selections were also chosen for their attractive tuber characteristics.

### Assessment of frost tolerance and tuber yield in the first clonal generation

In the season of 2011–2012, field trials were focused on verifying the consistency of frost tolerance responses for the 58 genotypes selected. In addition, these trials helped to better characterize tuber traits and estimate yield potential. Tubers harvested in the previous season were used as seeds that allowed more plants per genotype (up to 10 plants) to be assessed. Trials were done at the experimental station of *Kayra* in Cusco and the cold hardy cultivar *Locka* was used again as a comparative control.

The data collected at harvest consisted of tuber number and tuber weight per plant, which gave insights on the levels of yield variability among genotypes and allowed identification of genotypes with outstanding productivity. The levels of variation for yield among genotypes were very significant (p<0.001). The average tuber number per plant fluctuated between 1 and 54 (an average of 10), while total tuber weight per plant ranged from 1 to 860 g (with an average of 125 g). The regional cultivar *Locka* used as control averaged 12 and 307 g, for tuber number and weight, respectively. Based on the results of the evaluations, nine genotypes were identified with superior yield and were selected for additional validation of frost tolerance and agronomic traits in replicated field trials.

### Assessment of clonal selections

In the growing season of 2012–2013, the nine clones selected were evaluated in replicated field trials using randomized complete blocks. Each clone included 10 plants and up to four replications, all done at the experimental fields of the INIA station in *Salcedo* and *Illpa* in Puno. This is a region in the Altiplano where harsher climatic conditions are common particularly in *Illpa* where the average night temperature for the growing season is 3.4°C (ranging from −0.6°C to 5.7°C). According to the INDECI (Peruvian National Institute of Civil Defense), between the years 2003 and 2022, 1,168 frost events were recorded. The greatest number of frost events occurred in the province of Puno where Salcedo and Illpa are located. A comprehensive report for the historical occurrence and risks of frost in the Puno region is found in a report by Peru’s National Center for Disaster Risk Estimation, Prevention and Reduction (CENEPRED, 2022).

**Figure 2:**
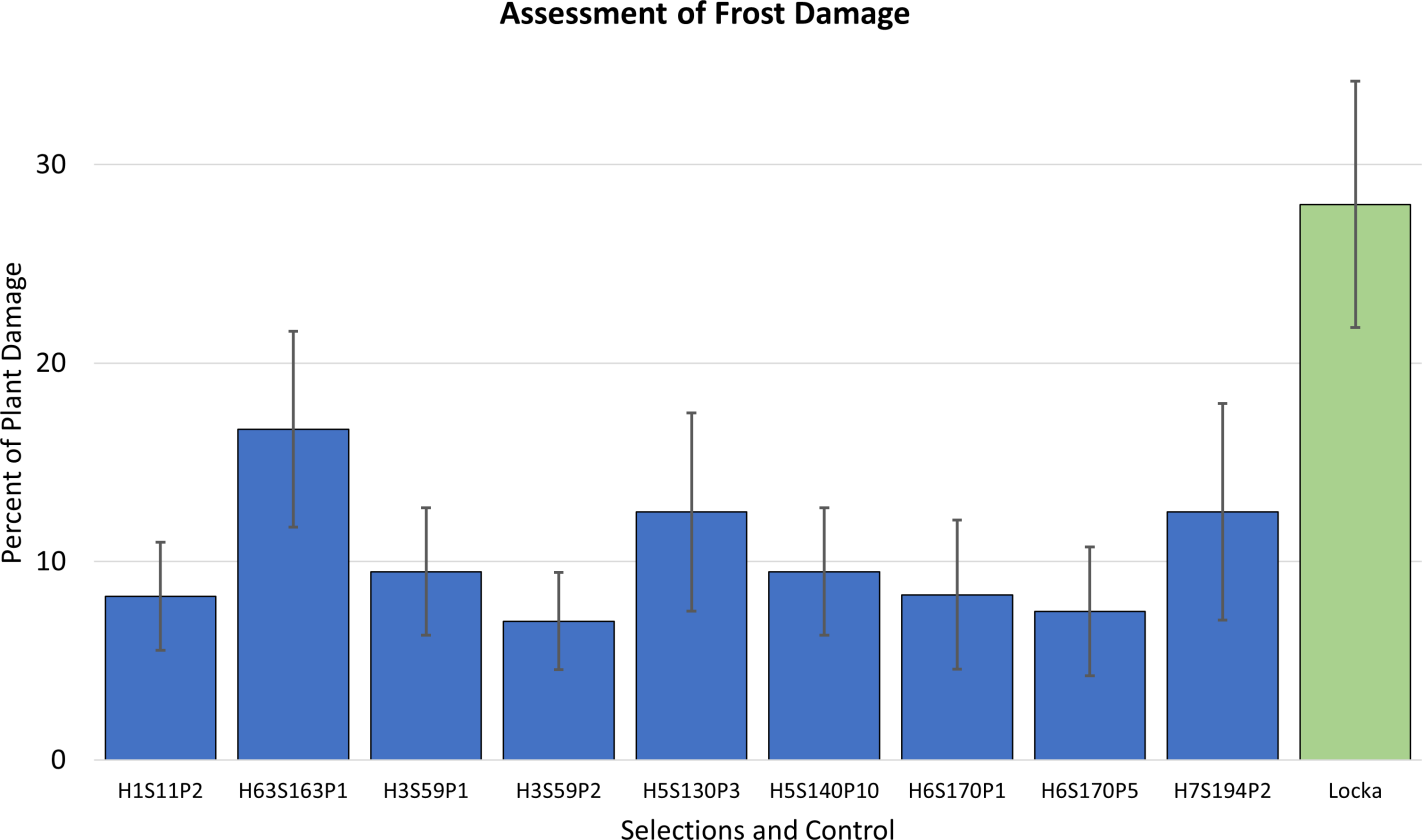
Assessment of the percent of plant damage that occurred after a frost for the nine advanced selections. Error bars represent standard deviation of the mean. The native cultivar *Locka* was included as control.

In-field evaluations of plant damage after frost confirmed that these nine selections exhibited outstanding frost tolerance. Six of them were revealed to be under 10% of plant damage; in comparison, cultivar *Locka* had a score of 28% (**Figure 2**).

**Figure 3:**
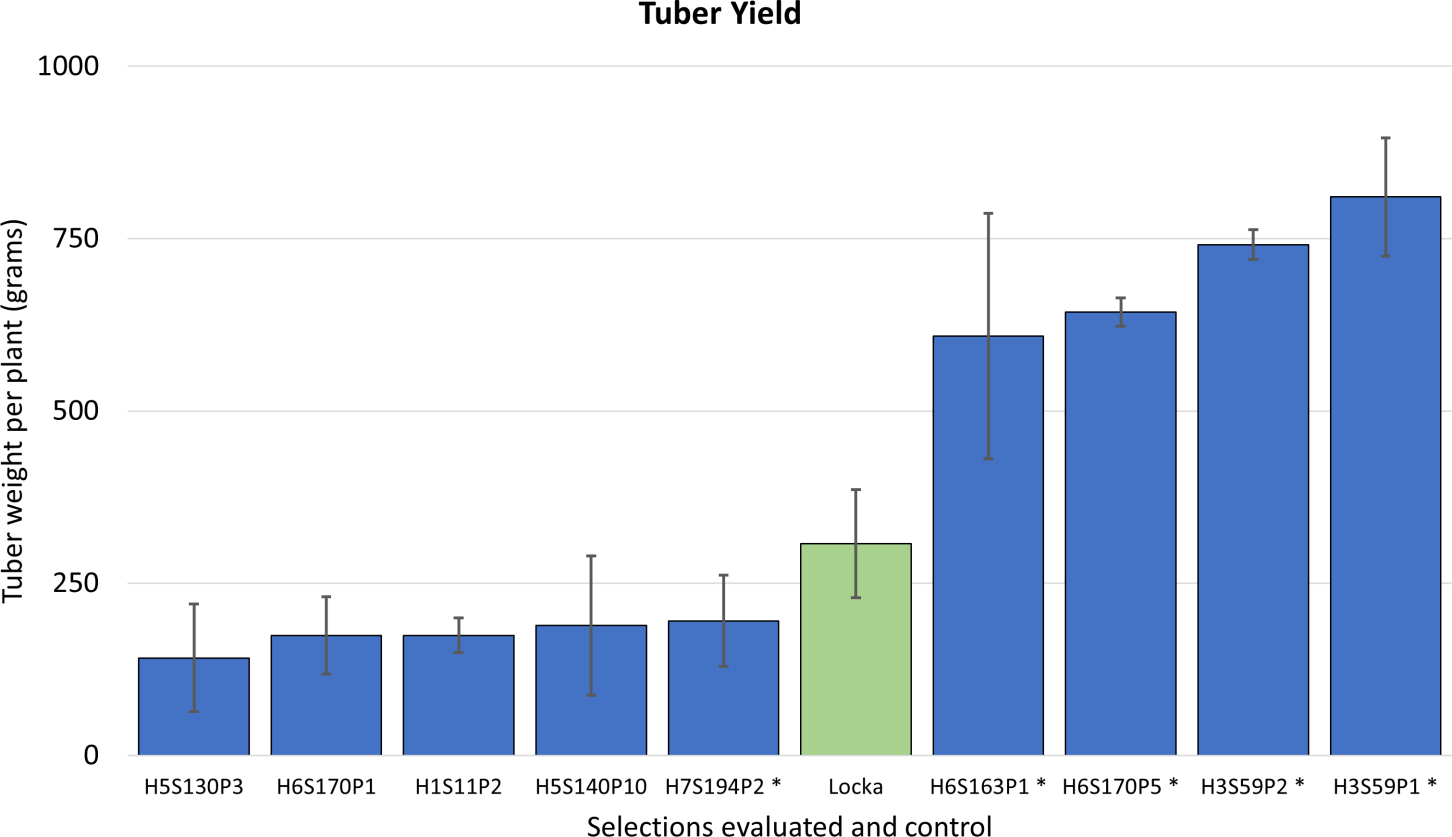
Yield (determined by average tuber weight per plant in grams) for the nine advanced selections evaluated in Salcedo and Illpa, Puno. Error bars represent standard deviations of the mean. Clones in asterisks are the five selections advancing to additional field trials and selection.

At harvest, it was found that some of these clones exhibited tuber yields that were significantly superior to the native cultivar used as the control (p<0.001) (**Figure 3**). This resulted in narrowing down selections to five clones, coded as H3S59P1, H6S163P1, H7S194P2, H6S170P5, and H3S59P2, that were rated as exceptional and chosen to be evaluated as potential cultivars (**Figure 3**). It is important to note that selection H7S194P2 was not particularly exceptional for frost tolerance and/or yield. However, farmers in the Andes/Altiplano do not always consider high yields as the main priority in selecting for a cultivar. Our experience of many years working with local farmers made us aware that varieties with attractive tuber features, including taste, are sometimes preferred over high yielders. In this case, the farmers considered that the clone H7S194P2 satisfied those attributes and advanced for additional testing.

### Comparison of selections to regional cultivars

Evaluations conducted in season 2014–2015, in replicated trials, included the five advanced clones selected in the previous season plus additional native potato cultivars common to the region. The cultivars included were *Lekecho*, *Mariva*, *Imilla Negra*, *Oke*, *Pucamama*, and *Imilla Blanca.* All are very common in stores and local farmers’ markets in the Altiplano and Southern Peru and have good levels of cold hardiness. The cultivar *Imilla Negra* is one of the most popular commercial cultivars in that region.

**Figure 4:**
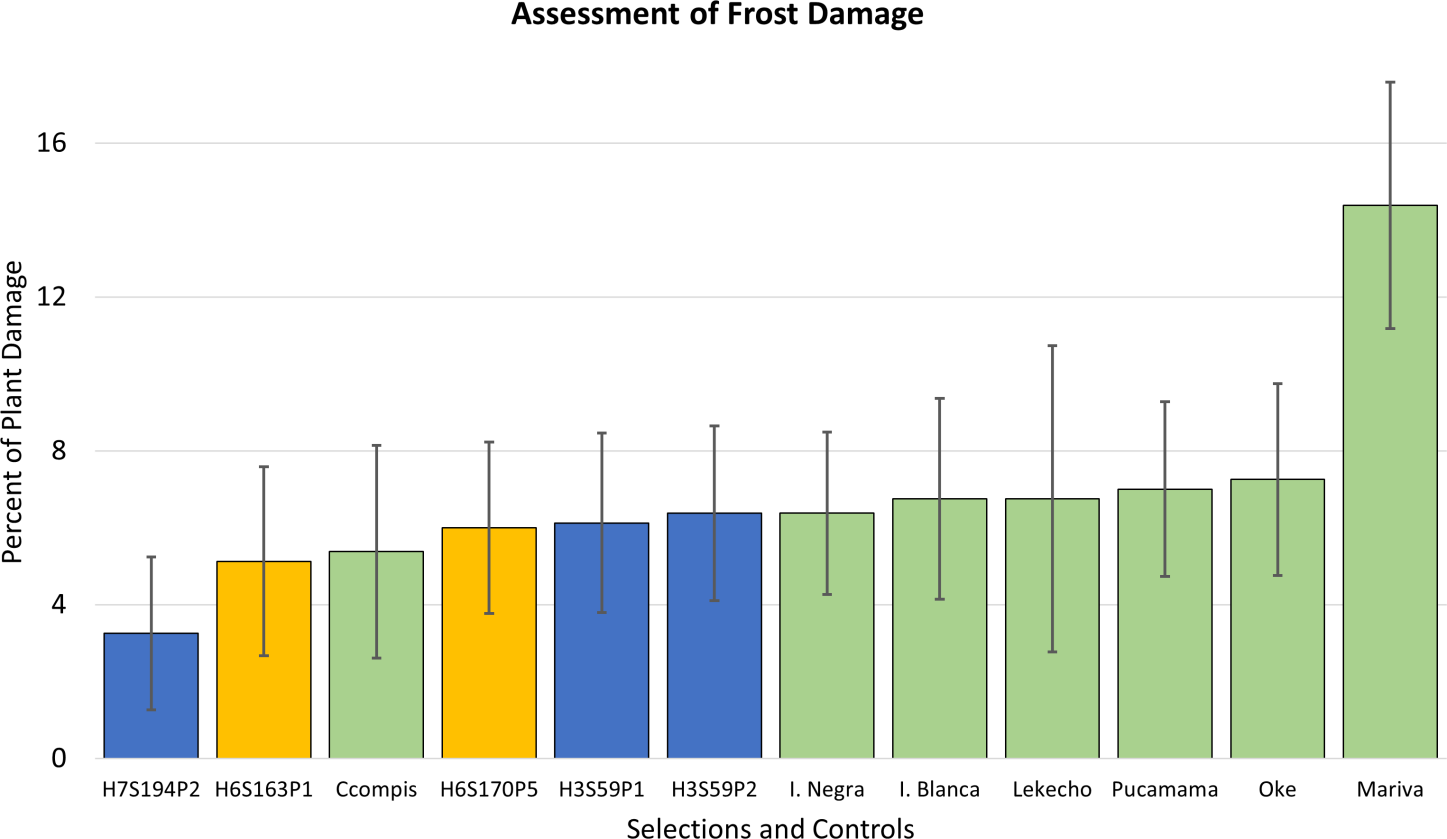
Frost damage scores are shown in percent of plant damage for five advanced frost-tolerant selections, which are compared to seven native cultivars from the region. Clones in yellow bars are the advanced selections that later resulted in cultivar releases. Error bars represent standard deviations of the mean.

The results of evaluations in the fields of Puno confirmed that the five advanced selections responded well to the climatic conditions of the region and exhibited excellent levels of frost tolerance. The scores of plant damage were in the range of 3% to 6%, and the traditional cultivars also exhibited good tolerance in the range of 7%–8% of plant damage except the cultivar *Mariva* with 14% (**Figure 4**).

At harvest, all advanced clones (except for H7S194P2) showed good tuber yields that were comparable to or better than those in the traditional cultivars. During the season, however, one of the top performers, H6S170P5, showed high levels of foliar spot disease and significant susceptibility to drought; for those reasons, this clone was excluded for further testing.

### Final years of selection and field trials for cultivar release

**Figure 5:**
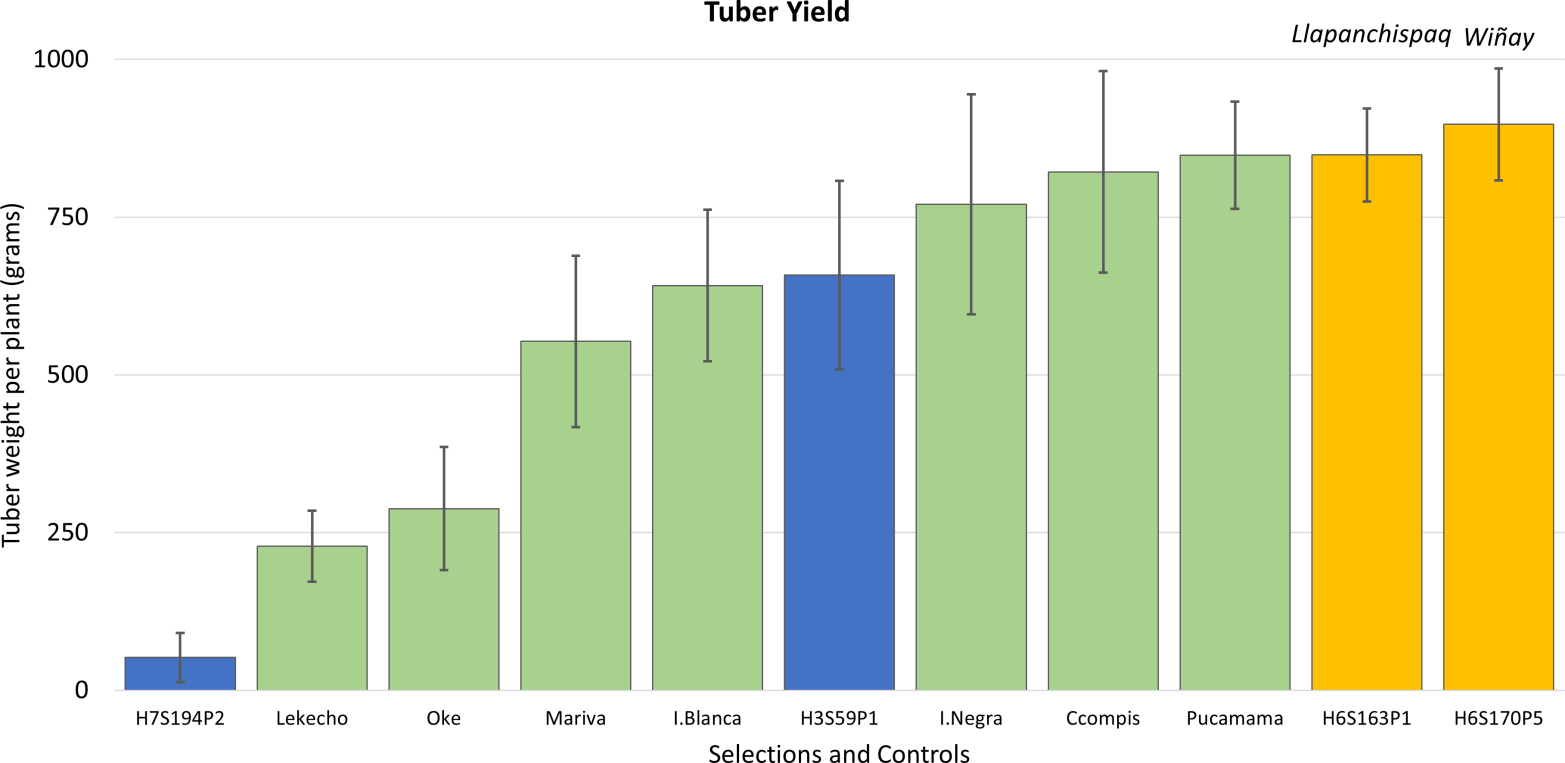
Yield (determined by tuber weight in grams per plant) from harvest data in four advanced selections and seven native cultivars evaluated in the fields of Salcedo and Illpa, Puno. Error bars are the standard deviations of the mean value. Yellow bars are the advanced clones later released as new cultivars; on top of the bar, their given released cultivar names are indicated.

In the next two seasons, which included the years 2015 to 2017, evaluations came down to four clones: H3S59P1, H6S163P1, H6S170P5, and H7S194P2. This 2-year assessment corroborated that tuber yields were excellent for these selections as they were comparable or better than some of the common cultivars (**Figure 5**). The average tuber yield per plant in all the selections combined was 601 g, and two clones, H6S170P5 and H6S163P1, exhibited the highest yields in the group with 897 and 849 g of tuber weight per plant, respectively. Therefore, it was decided to consider these two clones (H6S170P5 and H6S163P1) for potential release as new cultivars.

### Release of new potato cultivars for Peru

The field trials and surveys at the farmers’ communities to comply with Peru’s regulations for cultivar release, as indicated in INIA’s policies and guidelines, confirmed that clones H6S170P5 and H6S163P1 were acceptable in quality and productivity. Though glycoalkaloid levels in tubers were not measured, taste panels done among the farmers at the communities confirmed no perception of bitterness in either selection. As noted in the breeding scheme, these selections derived from non-bitter cultivars and hybrid lines (which were selected for reduced quantities of alkaloids). In addition, most of the wild *cmm* genome in the *cmm-tbr* hybrid was eliminated through backcrosses.

**Figure 6:**
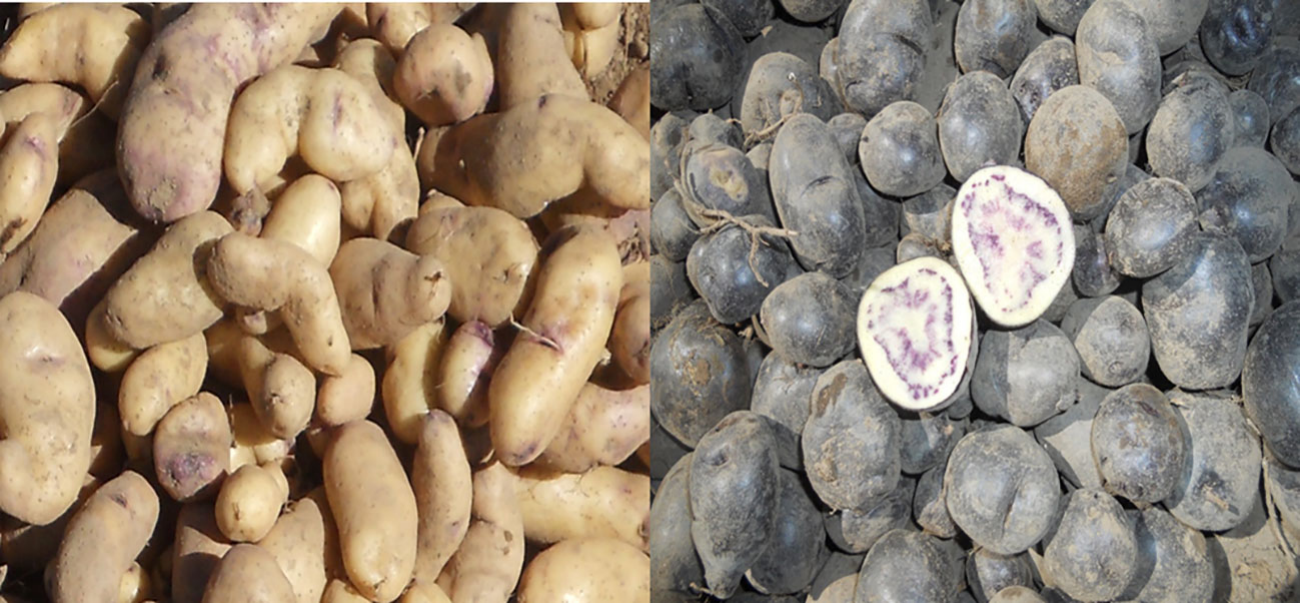
Pictures of the new potato cultivars with enhanced cold hardiness released in Peru: *Wiñay* H6S170P5 (on the left) and *Llapanchispa*q H6S163P1 (on the right).

**Figure 7:**
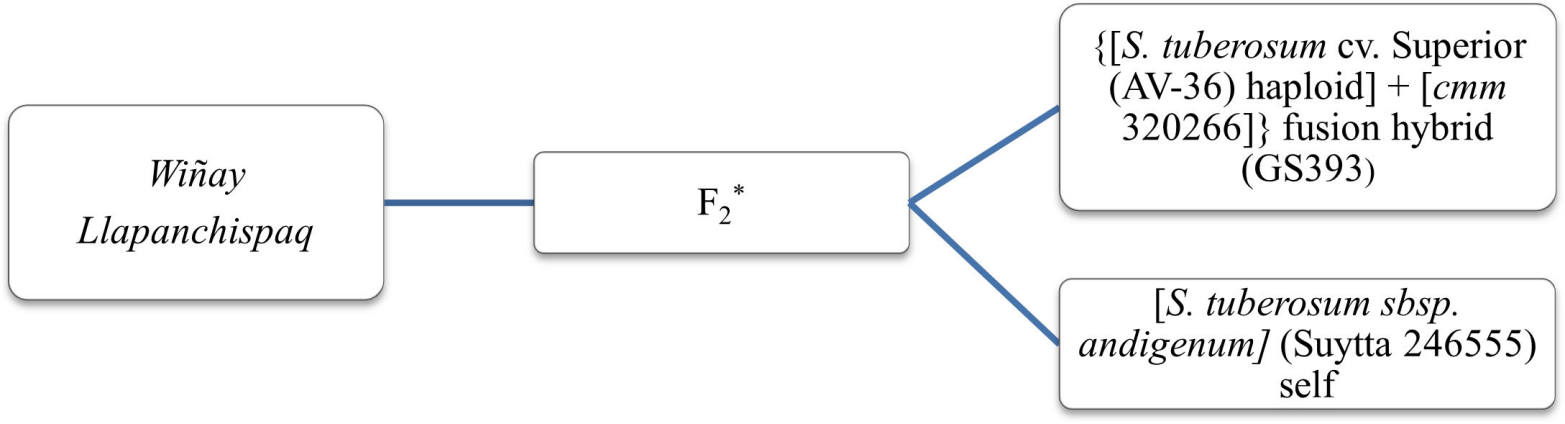
Pedigree of *Wiñay* (H6S170P5) and *Llapanchispaq* (H6S163P1), two new native potato varieties for Peru bred from a cross between female Peruvian native potato cultivar “*Suytta*” and male “*cmm + tbr* fusion hybrid GS393. *The F_1_ progenies from crosses were subsequently bulk intermated to generate the F_2_ populations that were sent to Peru.

The selections were then collectively accepted by the rural communities and, hence, approved as new cultivars for Peru. In November of 2018, clone H6S170P5 completed the required paperwork and was released as *INIA 330* with the cultivar name of *Wiñay* (a Quechua word meaning *to grow).* The first two letters of the cultivar name *Wi-* were also chosen to remember Wisconsin as the origin of the breeding line and the international collaboration. In November 2022, clone H6S163P1 also completed the requirements and was released as *INIA 334* with the name of *Llapanchispaq* (a Quechua word meaning *for all of us*) (**Figure 6**). The pedigree of these two new cultivars is shown in **Figure 7**.

## Discussion

An inspection of the potato seedlings after they were transplanted in the fields showed that the plants exhibited symptoms of environmental stress (as plants moved from steady conditions in the greenhouses to the natural fields in the highlands). Stresses included reduced water availability combined with lower humidity levels in the air and the colder temperatures common to high elevations of Andean environments. In fact, regardless of the time of the year, high elevation sites are characterized by unexpected cold spells at night and early morning. The farmers also indicated that the plants in two locations, *San Jose de Aymara* and *Aramachay*, endured periods of drought early in the season, which caused additional losses. The effects of drought can be very detrimental; more than 80% of the agriculture in the highlands of Peru is rainfed, and irrigation is not an alternative (Cruz et al., 2017). On the other hand, some fields experienced waterlogging stress after heavy rainfalls, especially in Cusco. All these situations illustrate that strong selective pressure, not just by frost, takes place in the Andes, which provides a glimpse of how challenging and unpredictable agriculture in the region can be.

During the growing seasons, frost episodes occurred at each location at different times (the potato growing season in the highlands of Central and Southern Peru runs from planting in October–November to harvest in May–June). The farmers living in the communities near the fields indicated that each frost they experienced was different in terms of its duration, temperature, and severity. For instance, a frost reported in *Ccorao* (one of the most frost-prone localities identified in the Cusco region) generated very low temperatures with the lowest point in the vicinity of −8°C. That variability and unpredictability of the conditions of each frost event underlines the fact that advances in research and breeding for frost tolerance are very challenging. Each frost occurrence has unique characteristics, so developing cold hardiness that can be expressed in a broader range of conditions could be the most advantageous.

Plants from the distinct breeding families showed different degrees of adaptation and fitness in the fields (in terms of the ability of the plant to grow and develop). As anticipated, the plants resembled the female parent, that is, they presented the morphological plant characteristics of native Peruvian potatoes of the cultivated species *adg*. This was sought in this project and was an important aspect in developing a breeding program there—it was critical to have breeding materials with the cultivar characteristics preferred and wanted by the local farmers. During the selection process, farmers were involved and shared their views, which were fully respected and used to make decisions on what genotypes to select. They mainly stressed the importance of selecting the attractive tuber characteristics favored in the region in terms of tuber shapes and colors. They also indicated their preference for plants with robust foliage.

**Figure 8:**
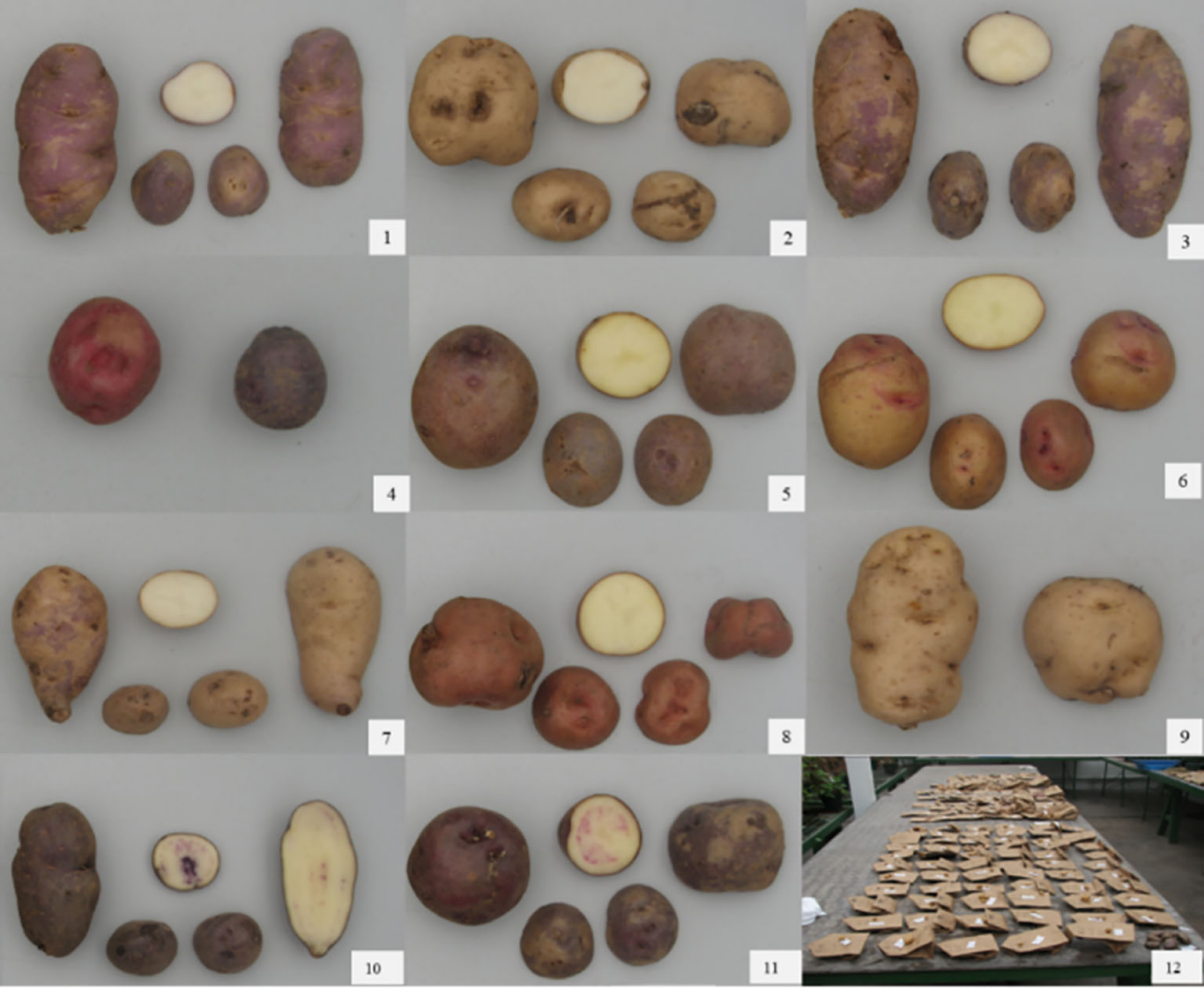
Some examples of tuber diversity for shape and color in the breeding lines assessed for this project. The clonal genotypes presented in the pictures are (1) H1S11P2, (2) H7S194P2, (3) H3S59P1, (4) H6S170P1, (5) H3S59P2, (6) H5S130P3, (7) H6S170P5, (8) H5S140P10, (9) H3S63P1, (10) H6S151P2, (11) H6S163P1, and (12) shows a picture of a large display of tubers that were used for selecting tuber features at the *Kayra* Experimental Station in Cusco.

All assessments done from seedlings to clonal generations were helpful to identify and confirm individuals with exceptional frost tolerance. Additional field trials of the advanced selections confirmed the consistency of frost hardiness at different years and locations. In the later trials, including more plants per each genotype not only helped to estimate yield potential but also to confirm expression of the tuber features, that is, shape, size, quality, and color (**Figure 8**). Tuber quality implied tubers with nice, healthy skin, resistant to bruising, and without significant external and internal defects like scab, browning, or hollow heart. In addition, the plants in the field were visually checked for signs of susceptibility to pests/diseases during the season, in particular, late blight (*Phytophtora infestans*) and the Andean potato weevil *(Premnotrypes* spp.), two of the most widespread biotic constraints affecting potato in the Andes. Altogether, we concluded that even though all the initial selections showed good levels of frost tolerance, not all of them were considered for more testing. Most of them (~84%) were found to not have enough yield and/or tuber quality to be included in further assessments. However, the few selections that reached the final stages of this breeding project were exceptional and outperformed many of the traditional cultivars included as controls. This finding is even more remarkable if we consider that the plants were exposed to the colder and tougher environmental conditions of the Altiplano, in the fields of *Salcedo* and *Illpa.* Under those circumstances, plant damage after frost was under 10%, which was an indication that genes for enhanced cold hardiness and acclimation capacity from *cmm* were likely transferred and expressed in the selections.

Another interesting result was that the two clones selected for cultivar release originated from the same breeding family coded as H6–. In this family, the female *adg* parent was a native cultivar named *Suytta* (which was donated to the USPG from Peru in 1958). The amount of data in the passport record of this collection at the USPG is limited. It reports that this accession was collected in the town of Pisac (near Cusco City), (probably) acquired at a local farmers’ market. About its characteristics, the records indicate that the tubers are round showing some blue or red and white colors and that plants do not produce many tubers, and yield is poor. The descriptions certainly conflict with what was observed in the field and agronomic evaluations, but we have to keep in mind that these new cultivars are also expressing traits of *tbr* and *cmm*.

**Figure 9:**
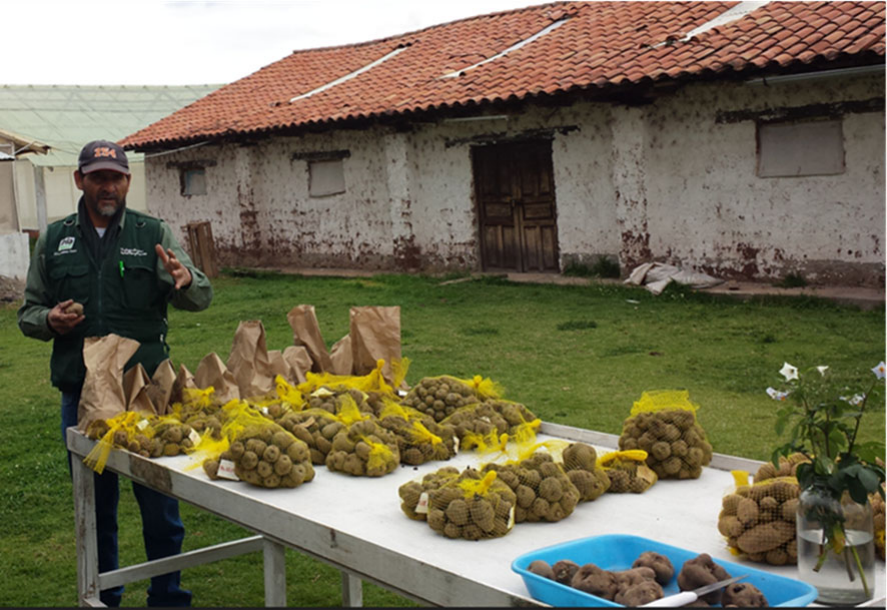
Cooperator Ladislao Palomino from the INIA-Cusco showing additional advanced native potato clones generated from the USPG breeding lines sent to Peru.

In a recent development, plants of *Wiñay* and *Llapanchispaq* growing in Puno in 2022 responded exceptionally well to a prolonged drought affecting the Altiplano region (J. Arcos, personal communication). Drought is also a major abiotic constraint common in the region and responsible for crop losses every year. Finding that the cultivars also have drought tolerance is excellent news and opens opportunities for enhancing food security and sustainability for the farmers in the Altiplano. Also, while this project was in the process of selecting *Wiñay* and *Llapanchispaq*, other additional promising clones shared as breeding stocks with other potato breeding and development efforts in Peru were evaluated (L. Palomino, personal communication). As a result, the potato breeding program at the INIA Cusco has reported several advanced clones that originated from the Wisconsin breeding lines, and that have excellent frost tolerance with potential to become new cultivars (**Figure 9**).

## Conclusions

The development and release of new cultivars *Wiñay* (H6S170P5) and *Llapanchispaq* (H6S163P1) are good examples of implementing a breeding project after effective assessment, use, and deployment of helpful diversity expressed in a wild potato relative. More significantly, this project properly aligns with global efforts aimed at solutions to counter climate change. These cultivars offer farmers opportunities for crop sustainability in regions where frost is serious and extensive. From a broader perspective, this derived from many years of scientific and technical advancement done at the USPG and UW. All led to the discovery of extreme cold hardiness in *cmm*, and later, to figure out that protoplast fusion and somatic hybridization can be used to overcome reproductive barriers in this species, and therefore, access its useful diversity for breeding.

In summary, this effectively integrated past and present accomplishments in research and in multi-institutional efforts in germplasm exchange, conservation, screening, and breeding. The results—two new palatable native cultivars for the Andes and the Altiplano with enhanced frost tolerance that out-yielded current popular varieties. This appears to be the first time that cold hardiness from *cmm* has been incorporated into released native Andean cultivars and that breeding lines created in the USA ended up as new potato cultivars for Peru. This approach proved that global cooperation was possible and successful in achieving the desired important goal of benefit sharing (i.e., *promoting the use of genetic resources for fair and equitable sharing of benefits from their use)* (SCB, 2011; Brink and van Hintum, 2020).

## Data availability statement

The raw data supporting the conclusions of this article will be made available by the authors, without undue reservation.

## Ethics statement

Written informed consent was obtained from the individual(s) for the publication of any identifiable images or data included in this article. If the image/s was/were reproduced from an existing publication.

## Author contributions

JA: Investigation, Supervision, Validation, Writing – original draft, Writing – review & editing. AR: Conceptualization, Funding acquisition, Investigation, Supervision, Writing – original draft, Writing – review & editing. JB: Conceptualization, Funding acquisition, Investigation, Supervision, Validation, Writing – original draft, Writing – review & editing. SV-S: Investigation, Methodology, Validation, Writing – review & editing. JP: Formal Analysis, Investigation, Supervision, Validation, Writing – original draft, Writing – review & editing. AS: Conceptualization, Investigation, Methodology, Validation, Writing – review & editing. RG: Conceptualization, Investigation, Supervision, Validation, Writing – review & editing. WR: Funding acquisition, Project administration, Resources, Supervision, Writing – review & editing. DE: Funding acquisition, Investigation, Resources, Supervision, Writing – review & editing.
